# Impact of Work Value Awareness on Self-Rated Physical Health of Rural-to-Urban Migrant Workers in China

**DOI:** 10.3390/healthcare9050505

**Published:** 2021-04-27

**Authors:** Fan Yang, Yao Jiang, Krishna P. Paudel

**Affiliations:** 1 Department of Labor and Social Security, School of Public Administration, Sichuan University, Chengdu 610065, China; yangfan1987@scu.edu.cn; 2 Department of Sociology, Zhou Enlai School of Government, Nankai University, Tianjin 300350, China; s20175706@stu.sicau.edu.cn; 3 Department of Agricultural Economics and Agribusiness, Louisiana State University (LSU) and LSU AgCenter, Baton Rouge, LA 70803, USA

**Keywords:** work value awareness, self-rated physical health, mental health, rural-to-urban migrant worker, China

## Abstract

We used data based on the China Labor-Force Dynamics Survey 2016 to examine the relationship between the work value awareness and the physical health of rural-to-urban migrant workers. The work value awareness was characterized by five dimensions: awareness of the emotional value, social value, respect value, ability value and interest value. Physical health was measured by a self-rated health assessment. The results from an IV-ordered probit model show that the awareness of work value has a statistically significant impact on the self-rated physical health of rural-to-urban migrant workers. The results also show that the impacts of work value awareness on rural-to-urban migrant workers’ physical health are heterogeneous to genders and ages. Mental health plays a mediating role between the awareness of work value and the physical health of rural-to-urban migrant workers. It is beneficial for rural-to-urban migrant workers to improve awareness of the value of work to enhance their physical health. The findings of this paper have policy implications for improving the health and welfare of Chinese rural-to-urban migrant workers as well as temporary migrants to cities in many developing countries.

## 1. Introduction

China was once known as the factory of the world. Rural-to-urban migrant workers with an education level at high school or below who have household registration in rural areas but currently work in cities have made significant contributions to this title [1]. A significant number of rural-to-urban migrant workers are engaged in manufacturing, construction, and service industries in cities. These workers have made great contributions to China’s economic growth and development. In 2019, the total number of rural-to-urban migrant workers in China reached 290.77 million, an increase of 2.41 million over the previous year, which is an increase of 0.8% [2]. Many rural-to-urban migrant workers can only work in the insecure informal employment sector, such as construction and food sectors in cities [3,4]. These workers face difficulty maintaining a decent living standard in urban areas [5].

Generally, awareness of work value refers to the workers’ awareness of the value of their work [6,7,8,9]. The awareness of the value of the work affects the work efficiency and job satisfaction of workers [10]. It may also affect workers’ welfare; especially their health, which is the focus of this paper.

Based on the theoretical analysis of the effect of work value awareness on the health of workers, this paper empirically analyzes the impact of work value awareness on the self-rated physical health of rural-to-urban migrant workers. We used the China Labor-Force Dynamics Survey 2016 (CLDS2016) data, which are nationally representative data. We discuss the mechanism by which work value awareness influences the self-rated physical health of Chinese rural-to-urban migrant workers. Further, we discuss the heterogeneous impacts of work value awareness on rural-to-urban migrant workers’ physical health in different gender and age groups. This study has policy implications for the work value awareness, health and welfare improvements of rural-to-urban migrant workers in China and migrant workers in other developing countries around the world.

Our main contributions are as follows: first, this study expands the influencing factors of workers’ health in a more elaborative way by examining the impact of work value awareness on the self-rated physical health of rural-to-urban migrant workers in China. Second, we address the endogeneity concern between work value awareness and physical health and identified the net effect of work value awareness on rural-to-urban migrant workers’ physical health. Third, by exploring the mediating effect of mental health, this paper provides a policy path of “work value awareness—mental health—physical health” to improve rural-to-urban migrant workers’ physical health. To the best of our knowledge, this is the first article that focuses on the impact of awareness of work value on the physical health of Chinese rural-to-urban migrant workers.

The remainder of this paper is organized as follows. In Section 2, we propose an analytical framework. In Section 3, we detail the methods used in the paper. We report the results in Section 4 and provide discussions in Section 5. Limitations are provided in Section 6, and conclusions with policy implications of the findings are described in Section 7.

## 2. Analytical Framework

**Figure 1:**
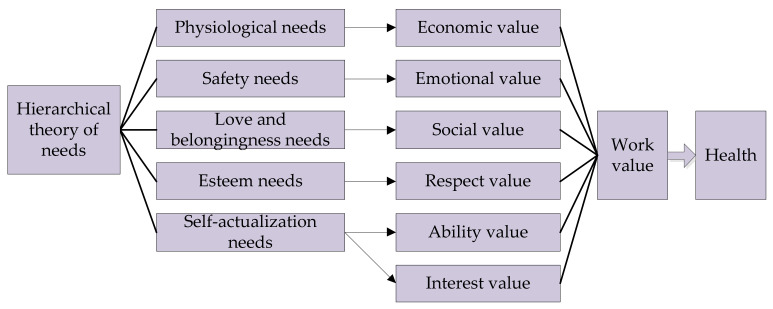
Analytical framework.

According to Maslow’s hierarchical theory of needs (HTN) [11], for workers, the value of a job should provide five needs: physiological, safety, love and belongingness, esteem, and self-actualization. Therefore, based on the HTN [11], this paper constructs an analytical framework of work value awareness (Figure 1). In this analytical framework, a job should provide six values to workers: economic, emotional, social, respect, ability, and interest. Specifically, the economic value mainly provides the physiological needs; the emotional value primarily matches the safety needs; the social value mostly meets the love and belongingness needs; the respect value mainly meets the esteem needs; ability and interest values primarily set up corresponding relationships with the self-actualization needs.

The economic value of a job is that it provides a material basis for an individual’s survival and development. Therefore, we can match the economic value of a job with the physiological needs in the HTN. Reliable income flow and wealth accumulation allow individuals to have a healthy lifestyle and pay for health care services when needed [12].

In the HTN, safety needs are interpreted as people’s needs for stability, security, protection, order, and freedom from fear and anxiety [11]. Accordingly, we argue that the safety needs of a job for workers are reflected on the basis that the job can bring financial and emotional stability. Therefore, the emotional value of work matches the safety needs. Previous studies have confirmed that stable emotions are conducive to individual physical and mental health [13,14]. In terms of physical health, a review study has shown that positive emotions are good for cardiovascular health [13]. In terms of mental health, emotion is not only a key symbol of mental health, but also affects mental health. Good emotions lead to healthy psychology [14]. Therefore, if a job brings stable emotions to workers, it may help them to maintain health.

The social value of work mostly meets the love and belongingness needs for workers, which are the needs for interpersonal relationships including friendship [11]. Jobs provide a chance for workers to meet friends on the same wavelength, and thus affects personal health [15]. As an imperative form of social support, a like-minded friend possesses significant positive influences on individual health via effectively preventing individual harmful health behaviors, such as suicide and self-harming [16], and help overcome distressed emotions such as depression and loneliness [17]. Additionally, friends are crucial links of individual social life-networks and sources of social capital accumulation [18,19]. Social life-networks and accumulated social capital help individuals find a higher salary job that relieves economic distress and gets rid of destitution and obtains further better healthcare to maintain health [20,21]. For rural-to-urban migrant workers, the social value of work may or may not be there as they have to leave their familiar setting and settle in unknown areas, lacking the support system they once were familiar with [22]. This situation may be harmful to their health.

The respect value of work mainly meets the esteem needs of workers. On the plus side of valued work is the gain of respect from others. A job that others respect not only can be conducive to keeping workers delighted and decent, but spur individuals to work harder than before [23,24]. Thus, work efficiency and performance can be improved by these social esteems [25]. High work efficiency and performance also can form more respect from others. Therefore, a virtuous circle is formed: being respected—working harder—achieving good working results—getting more respect. In this cycle, being respected makes individuals feel self-worthy in society, which is beneficial to individual mental health. Meanwhile, physical health and mental health are intimately correlated with each other [26]. The work value of gaining respect may not affect individual physical health directly, but it mainly affects physical health through mental states indirectly. However, due to being at the bottom of the society [27], rural-to-urban migrant workers receieve little respect from others both in the work and social environment; on the contrary, they receive more discrimination from the surroundings [28]. As the previous analysis, not receiving enough respect may have adverse effects on the health of rural-to-urban migrant workers.

The ability and interest values of work are primarily set up corresponding relationships with the self-actualization needs for workers. For the ability value, giving full considerations to one’s personal work ability in a job can reduce the sense of frustration brought by the mismatch of personal ability and work position, and thus strengthen the possibility of workers’ self-actualization in their work position, which enhances workers’ mental health [29,30]. Moreover, the ability value is positively associated with workers’ physical health [31]. When workers’ ability values are brought into full consideration, they may adopt and maintain healthy lifestyles such as taking physical activity, and giving up smoking and alcohol [32,33]. A cross-sectional survey has shown that the employed spend less time sitting idle and have healthier lifestyles than the unemployed [34]. The contributions of rural-to-urban migrants to develop urban areas are well recognized in China. Therefore, how the rural-to-urban migrant workers’ self-actualization in this contribution affects their health should receive further attention.

Interest is the best teacher to motivate individuals to do what they like to do. An individual is attracted by his preferred activity or thing. Therefore, interest is based on needs, and individuals obtain psychological satisfaction from their own interests or hobbies [35]. As such, it is helpful for individuals’ mental health to engage in the work they are interested. In terms of empirical research, a study of 605 Chinese adolescents has shown that social interest is a significant factor affecting their mental health [36]. However, the work of rural-to-urban migrant workers is generally low-end and repetitive, rather than interest-oriented [37], which may negatively affect their mental and physical health.

From the above description, it can be cautiously concluded that workers’ awareness of the value of their work may have a positive or negative effect on their physical and mental health. However, if accurate results are to be obtained, empirical tests are needed. Health is a vital human capital, which is the basis and premise of other forms of human capital, such as education and professional skills [16]. It is significant for the survival and development of individuals and it is a right that workers should have. At the same time, recognizing the value of the work they are engaged in is needed for individuals to accept their work, gain self-confidence and satisfaction from their work. Therefore, it is of practical significance to study the effect of work value awareness on workers’ health.

The general public in China does not have a full understanding of rural-to-urban migrant workers’ livelihood situations. They do recognize the contribution of rural-to-urban migrant workers to the economic development of the whole country [38]. At the same time, rural-to-urban migrant workers are labeled as ignorant and backward, and they are regarded as a group of people eliminated by the times [39]. Rural-to-urban migrant workers themselves often live in self-denial; they hold that they are far from the success that the public has defined [40]. These rural-to-urban migrant workers do not think their work is something to be proud of [41]. Generally speaking, rural-to-urban migrant workers’ awareness of the value or meaning of their work is low, which may have significant negative influences on their health.

## 3. Methods

### 3.1. Data

The dataset used in this article comes from the CLDS2016, which was carried out by Sun Yat-sen University in 2016. It is one of the largest and most authoritative datasets in China that collected information about the education experience, work status, entrepreneurial experience, and health status of Chinese workers (rural workers, urban workers, and rural-to-urban migrants). The dataset includes workers from 29 provincial administrative units which ensures the national representativeness of the samples (information is not collected from Hong Kong, Macao, Taiwan, Tibet, and Hainan). We focus on only rural-to-urban migrant workers in this study. The number of observations used in this study is 908.

### 3.2. Measurements

#### 3.2.1. Dependent Variable

This paper examines the impact of rural-to-urban migrant workers’ awareness of work value on their physical health. Therefore, the dependent variable of this paper is the rural-to-urban migrant workers’ physical health. We used self-rated health to measure the physical health of the subjects. Respondents were asked, “How do you evaluate your current health status?” The answer was measured by a five-point Likert scale ranging from “1” to “5”. An answer of “very unhealthy” was coded as “1”, “somewhat unhealthy” was coded as “2”, “normal” was coded as “3”, “somewhat healthy” was coded as “4”, and “very healthy” was coded as “5”.

#### 3.2.2. Explanatory Variables

We mainly focussed on how migrant workers’ awareness of emotional value, social value, respect value, ability value and interest value of the works they are engaged in affect their health status.

For the emotional value awareness, respondents were asked, “Do you agree with the statement that my current job can make me feel at ease?” Similarly, for the social value awareness, respondents were asked, “Do you agree with the statement that my current job can make me get to know more people?” For the respect value awareness, respondents were asked, “Do you agree with the statement that my current job can make me get respect from other people?” Further, for the ability value awareness, respondents were asked, “Do you agree with the statement that my current job can give my ability to full play?” In addition, for the interest value awareness, respondents were asked, “Do you agree with the statement that my current job can satisfy my interest?” The answers for the above five questions were all measured by a five-point Likert scale ranging from “1” to “5”. An answer of “strongly agree” was coded as “1”, “agree” was coded as “2”, “normal” was coded as “3”, “disagree” was coded as “4”, and “strongly disagree” was coded as “5”. This means that the larger the number, the smaller the work value awareness.

To obtain the total work value awareness, the awareness of emotional value, social value, respect value, ability value, and interest value of migrant workers’ work were aggregated. We tested the validity of the above five work value awarenesses. Polychoric correlation coefficient results show that the minimum correlation coefficient among the variables of work value awareness is 0.3543 (Table 1). It indicates that the five dimensions of work value awareness have good validity to measure the work value awareness of rural-to-urban migrant workers.

#### 3.2.3. Mediating Variable

**Figure 2:**
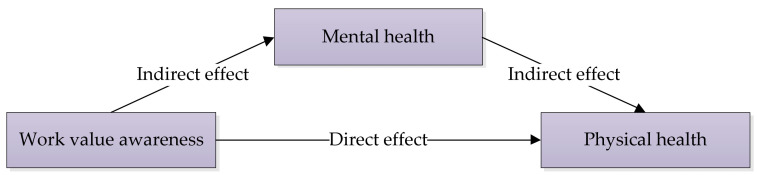
Mental health as a mediating role in the impact of work value awareness on physical health.

As described in the introduction section, most of the time, the awareness of work value has an impact on mental health firstly, and then on physical health. In this process, mental health plays a mediating role (Figure 2). Therefore, we chose mental health as the mediating variable of the impact of work value awareness on rural-to-urban migrant workers’ physical health. We used the Center for Epidemiological Studies Depression Scale (CES-D) to measure the respondents’ mental health (Appendix A). CES-D is one of the most widely used tools for assessing depression and mental health, developed initially by Lenore Radloff of Utah State University [42]. The scale contains 20 items, each scored from “1” to “4” (“1” = rarely or none of the time ( < 1 day), “2” = some or a little of the time (1–2 days), “3” = occasionally or a moderate amount of the time (3–4 days), “4” = most or all of the time (5–7 days)) on a Likert scale except questions 4, 8, 12, and 16. For questions 4, 8, 12, and 16, the scoring is exactly the same except that it is reversed: “Most or all of the time (5–7 days)” is scored 1 point, “Rarely or none of the time ( < 1 day)” is scored 4 points, etc. The total score ranges from “20” to “80”. Roughly speaking, the higher the score is, the more serious the depression level is and the worse the mental health is.

#### 3.2.4. Control Variables

To obtain the effects of work value awareness on rural-to-urban migrant workers’ physical health, we controlled other factors which may affect workers’ physical health, including gender, age, education, marriage, religion, income, lifestyle (smoking and drinking), and the regional effect.

Gender: The health of females and males has shown different results [43]. Females often have better physical health status than males but worse mental health [3]. Therefore, in this paper, we controlled gender, which is a dummy variable (1 = male, 0 = female).

Age: It is recognized that people’s health status declines as they age [44]. We measured age as a continuous variable.

Education: Education has a significant influence on individuals’ physical health and mental health via healthcare accessibility [44,45]. We used education as a continuous variable. 

Marital status: With regard to marital status, marriage is found to be a vital way to alleviate individual health problems [46]. Married people often experience less health distress compared to a single person [16]. As such, this paper divides the marital status into “single”, “married”, “divorced”, and “widowed”, and we measured it as a dummy variable. 

Religion: A previous study has shown that some religious people in China have an attitude of fatalism to disease, which may cause their health to deteriorate [47]. We categorized religion to “western religion”, “eastern religion”, and “no religion.”

Income: Studies have consistently documented that, in general, people with a higher income are healthier than those with a lower income [48]. Therefore, income as an important variable affecting individual health. Rural-to-urban migrant workers’ income in this paper is a continuous variable and referred to the total income of workers in 2015.

Smoking/drinking: Multiple studies on the factors affecting individual health provide evidence concerning damaging health habits such as smoking and drinking [33,34]. In this paper, smoking and drinking both are a dummy variable, and were measured by asking the questions, “Do you have the habit of smoking/drinking?” The answer with “1” represents “yes” and “0” otherwise.

Regional effects: We controlled the regional effect, and region was a dummy variable measured by the provinces where respondents are located.

### 3.3. Data Analysis Strategy

Our dependent variable (self-rated physical health status) was a discrete variable, so we used an ordered probit regression model [49]. Generally, an ordered probit empirical model is formulated as:

(1) $$y^{*}=\beta x+\gamma Z+\varepsilon$$

where, $y^{*}$ is the dependent variable, representing the self-rated physical health of rural-to-urban migrant workers. x represents the work value awareness of rural-to-urban migrant workers. Z represents control variables that may affect the physical health of rural-to-urban migrant workers. β and γ represent the coefficient vectors. ε is the error item.

We do not observe $y^{*}$; however, we observe y. Assuming the selection rule of y:

(2) $$y=\left\{\begin{array}{c} =1\;\;\;\;\;\;\;\;if\;\;\;\;\;\;\;y^{*}\leq 1\\ =2\;\;\;\;\;\;\;\;\;1<y^{*}\leq \mu_{1}\\ =3\;\;\;\;\;\;\;\mu_{1}<y^{*}\leq \mu_{2}\\ \cdots \;\;\;\;\;\;\;\;\;\;\;\;\;\;\;\;\;\;\;\cdots \;\;\;\;\;\;\\ =J\;\;\;\;\;\;\;\;\;\;\;\;\;\;\;y^{*}\geq \mu_{J-2} \end{array}\right.$$

where, $\mu_{1}<\mu_{2}<\cdots \leq \mu_{J-2}$ are unknown parameters that must be estimated along with β and γ. Meanwhile, in this paper, the endogenous problems between rural-to-urban migrant workers’ self-rated physical health and their work value awareness may present in the ordered probit regression model. The reasons can be stated in the following paragraphs.

First, rural-to-urban migrant workers’ work value awareness is an endogenous selection rather than a random selection. Rural-to-urban migrant workers’ work value awareness is the result of their self-selection, which means the work value awareness may be affected by other unobserved factors. Meanwhile, these unobservable factors have impacts on rural-to-urban migrant workers’ self-rated physical health. Therefore, the selection bias associated with rural-to-urban migrant workers’ work value awareness leads to an endogeneity concern.

Second, rural-to-urban migrant workers’ work value awareness and their self-rated physical health may have a mutually causal relationship. As aforementioned, rural-to-urban migrant workers’ work value awareness can impact their physical health via their mental health status. However, rural-to-urban migrant workers’ physical health also can affect their work value awareness. Generally, workers with a good physical health may have a more positive attitude and awareness of their work compared with those with a bad physical health [50].

Third, because the rural-to-urban migrant workers’ self-rated physical health is a subjective judgment, its measurement may have bias, which also may result in an endogeneity concern.

Fourth, the endogenous problem also may be caused by some factors unobserved in the model. Although the general factors affecting rural-to-urban migrant workers’ self-rated physical health have been controlled in the model, there may still be missing variables not included in the model.

Therefore, this paper uses an instrumental variable (IV) ordered probit model for estimation to solve the endogenous problems, lessen the estimation bias of results, and strengthen the results’ reliability.

Based on the peer effect [51], this paper applies the mean value of rural-to-urban migrant workers’ work value awareness in the same community except for rural-to-urban migrant worker i as the IV. Theoretically, the mean value of rural-to-urban migrant workers’ work value awareness in the same community except for rural-to-urban migrant worker i is a qualified IV, and it meets the two requirements of IV: relevance and exclusion. The work value awareness of rural-to-urban migrant workers who come from a same community may be affected by each other, resulting in the similar work value awareness. It indicates the IV we choose is closely correlated to the explanatory variable, rural-to-urban migrant workers’ work value awareness. Further, the mean value of rural-to-urban migrant workers’ work value awareness in the same community except for rural-to-urban migrant workers i does not have a direct association with workers’ self-rated physical health. Therefore, it qualifies for the requirement of exclusion.

The IV we applied was conducted by a diagnostic test. The F-value is far greater than 10. This meant that the IV, the mean value of rural-to-urban migrant workers’ work value awareness in the same community except for rural-to-urban migrant workers i, was a strong IV. Additionally, in the first stage regression, high R^2^ was found. When we regressed IV to the dependent variable, the coefficient was found to be not significant. The above results indicate that the IV we proposed satisfies the conditions of relevance and excludability, and the results estimated in this paper are reliable.

Moreover, to elicit the influential mechanism of rural-to-urban migrant workers’ work value awareness on their self-rated physical health and test the mediating effect of mental health, we used bootstrapping. The basic principle for the bootstrap approach is that the standard error estimation and confidence interval (CI) which are calculated based on the assumption of a normal distribution, will usually be imprecise because the indirect effect estimates generally do not follow a normal distribution [52].

## 4. Results

### 4.1. Descriptive Analysis

We find that the proportion of rural-to-urban migrant workers engaged in the tertiary industry is 51%, and that of the secondary industry is 48.6% [2]. The distribution of the samples is shown in Table 2. With regard to the dependent variable, self-rated physical health, the average value of rural-to-urban migrant workers’ self-rated health is 3.82, ranging from 1 to 5. The explanatory variable, the work value awareness, ranges from 5 to 25, with a mean value of 13.03. For the itemized work value awareness, the average values of emotional value, social value, respect value, ability value, and interest value awareness are 2.38, 2.63, 2.59, 2.64, and 2.79, respectively, and all the itemized work value awareness range from 1 to 5. The mean value of the mediating variable, mental health, is 25.79, with a minimum of 20 and a maximum of 76.

With regard to the control variables, out of the total samples, 52.86% of the rural-to-urban migrant workers are male. The age of the sampled rural-to-urban migrant workers ranges from 20 to 65 years old, with a mean value of 43 years. The average education level of the samples is 8.56 years, with a minimum of 0 years (illiteracy) and a maximum of 12 years (high school). The yearly income of rural-to-urban migrant workers had a mean value of CNY 41423.56 in 2015. We find that 15.53% of the respondents are single, 83.37% are married, 0.88% are divorced, and 0.22% are widowed. The percentage of rural-to-urban migrants who indicated not following any religions is 89.98%, having western religions is 1.98%, and having eastern religions is 8.04%. In terms of lifestyles, 31.50% of the respondents smoke and 24.23% of the respondents drink alcohol.

To estimate the results accurately, we conducted a multicollinearity test for all involved variables. The results of the multicollinearity test display that the variance inflation factor (VIF) has a mean value of 1.29, with the maximum value of 1.79, and the minimum value of 1.02. This indicates that there is no serious multicollinearity among variables in our models.

### 4.2. Benchmark Regression

The ordered probit model was employed as the benchmark regression to estimate the influences of rural-to-urban migrant workers’ work value awareness on their self-rated physical health. The results are shown in column (1)—(3) of Table 3. Column (1) in Table 3 displays the influence of rural-to-urban migrant workers’ work value awareness on their self-rated physical health, which is estimated without any control variables. The results in column (1) show that the rural-to-urban migrant workers’ work value awareness significantly influences their self-rated physical health. The results reported in column (2) show similar estimation outcomes as shown in column (1) after controlling regional effects. The results in column (3) present that the rural-to-urban migrant workers’ work value awareness still has a statistically significant correlation with self-rated physical health at a 1% significance level when the regional effects and respondents’ characteristics are controlled. The above results indicate that the more strongly rural-to-urban migrant workers recognize the value of the work they are engaged in, the healthier results they have rated for their physical health.

With regard to the results of control variables in column (3) of Table 3, the effects of the control variables are mostly in line with our theoretical expectations and consistent with previous studies. With the increment of age, the self-rated physical health declines. Marital status does have significant influences on rural-to-urban migrant workers’ self-rated physical health. Further, rural-to-urban migrant workers’ self-rated physical health is associated with their mental health significantly, which means the better mental health rural-to-urban migrant workers have, the higher the possibility they rate their own physical health as good.

To conclude, the results of the ordered probit model align with the aforementioned theoretical analysis. Work value awareness can increase the probability that rural-to-urban migrant workers judge their physical health outcome as healthy and decrease the probability that those judge their health status as unhealthy. However, the endogenous concern is not addressed by the benchmark regression model. Therefore, in the next section, we describe the results obtained from an IV-ordered probit model to further explore the relationship between rural-to-urban migrant workers’ work value awareness and their self-rated physical health.

### 4.3. Results from an IV-Ordered Probit Model

To address the endogenous problems caused by selection bias, reverse causality, measurement bias, and missing variables, an IV-ordered probit model is employed for estimation [53]. After controlling the endogeneity, the results presented in column (4)–(5) of Table 3 show that the impact of work value awareness on rural-to-urban migrant workers’ self-rated physical health is still statistically significant.

After adding the IV into the model, the impacts of control variables in column (5) of Table 3 also are consistent with the results in column (1)–(3) of Table 3. The age, marital status, and mental health are still correlated with the self-rated physical health of rural-to-urban migrant workers significantly. Specifically, with the increment of age, the self-rated physical health declines. Marital status does have significant influences on rural-to-urban migrant workers’ self-rated physical health. Further, rural-to-urban migrant workers’ self-rated physical health is associated with their mental health significantly, which means the better mental health rural-to-urban migrant workers have, the higher the possibility they rate their own physical health as good.

To conclude, the results of the IV-ordered probit model align with the aforementioned theoretical analysis; that is, work value awareness can increase the probability that rural-to-urban migrant workers judge their physical health outcome as healthy and decrease the probability that those judge their health status as unhealthy.

### 4.4. Heterogeneous Impact Analysis

We further explore the heterogeneous impacts of work value awareness on rural-to-urban migrant workers’ self-rated physical health in different gender and age groups. The full sample is grouped into four sub-groups: male and female, the younger generation and older generation. The rural-to-urban migrant workers born after 1980s are named the younger generation rural-to-urban migrant workers [54]. The sub-sample regression models are also estimated by using an IV-ordered probit model. The results are reported in Table 4.

The results in Table 4 show that the impacts of work value awareness on rural-to-urban migrant workers’ self-rated physical health are heterogeneous. For the gender groups, work value awareness of male rural-to-urban migrant workers is significant at the 10% level. The results indicate that the self-rated physical health of male rural-to-urban migrant workers are more easily impacted by work value awareness, compared with female workers.

Further, for the age groups, the older generation rural-to-urban migrant workers’ work value awareness has a significant correlation with self-rated physical health. However, the coefficient of the younger generation’s work value awareness is not significant. It means that compared with the younger generation rural-to-urban migrant workers, the self-rated physical health of the older generation rural-to-urban migrant workers has a higher possibility of being impacted by work value awareness.

### 4.5. Mechanism Analysis

As stated in the introduction section, we assume that work value awareness impacts rural-to-urban migrant workers’ self-rated physical health via their mental health status. Therefore, bootstrap estimation was employed to test the mediating effect of rural-to-urban migrant workers’ mental health.

The bootstrap samples were drawn 500 times to test the mediating effect. The estimated effects and 95% CIs of the direct and indirect effects are shown in Table 5. It can be observed that the direct effect, work value awareness → physical health, is significant at 5%, and the 95% CIs do not overlap zero. Additionally, the indirect effect, work value awareness → mental health → physical health, is significant at 1%, and the 95% CIs do not overlap zero. To sum up, the rural-to-urban migrant workers’ mental health plays a mediating role in the impact of work value awareness on rural-to-urban migrant workers’ self-rated physical health.

## 5. Discussion

We examined the relationship between the awareness of work value of Chinese rural-to-urban migrant workers and their self-rated physical health. We found that, in general, the stronger the awareness of work value, the better the self-rated physical health of rural-to-urban migrant workers, which is consistent with the theoretical framework of this paper. Therefore, the awareness of work value has a significant impact on rural-to-urban migrant workers’ self-rated physical health. As such, improving rural-to-urban migrant workers’ work value awareness is conducive to their self-rated physical health, and how to improve the awareness of work value requires additional attention.

We provide some suggestions for reference and further verification. To improve work value awareness, we should focus on three aspects: rural-to-urban migrant workers, enterprises, and local governments. For rural-to-urban migrant workers, the first step is to find the work they are interested in as much as possible. As pointed out in the previous theoretical analysis, interest is a vital driving force to motivate individuals to devote themselves to the work they are doing. For individuals, doing the work they are interested in is an easy way to achieve good work results and performance, and gain the recognition of the employers and colleagues, so as to establish the recognition of the value of their work [55].

For enterprises, although most rural-to-urban migrant workers work from rural areas to cities mainly for their livelihoods, and interest is not the first thing they consider when looking for a job, the importance of interest in their awareness of the value of their work cannot be ignored. When recruiting rural-to-urban migrant workers, enterprises should provide them with jobs that interest them as much as possible. In order to achieve this goal, enterprises can take the way of job rotation to help rural-to-urban migrant workers explore the work positions which interest them.

For local governments, efforts should be made so that the public correctly recognize the value of rural-to-urban migrants’ work. The governments should solicit help from media and non-governmental organizations to educate the public about importance of all work, irrespective of professions. It is difficult to eliminate prejudice, but it should not be allowed to freely multiply.

From the perspective of gender, male rural-to-urban migrant workers’ awareness of work value significantly impacts their self-rated physical health, while female rural-to-urban migrant workers’ awareness of work value has no obvious impact on their self-rated physical health. The possible reason is that there are differences in awareness of work value between male and female rural-to-urban migrant workers. Male rural-to-urban migrant workers pay more attention to the value of work than female rural-to-urban migrant workers. However, the overall underestimation of the value of rural-to-urban migrant workers’ work in society has hurt the psychology of male rural-to-urban migrant workers and further affected their physical health. Therefore, social policy should pay more attention to male rural-to-urban migrant workers’ awareness of work value, and constantly improve their awareness of work value.

In China, women have traditionally relied on men, which is still common among the lower economic echelon of society. Female rural-to-urban migrant workers mostly follow their husbands to work in cities from rural areas. They take concerted action with their husband in the cities, and most of the time, assist in their husband’s work. They care less about the value of their work than male rural-to-urban migrant workers. However, this does not mean that the awareness of work value is not important for female rural-to-urban migrant workers. On the contrary, losing the sense of the value of work is terrible. It is easy for individuals to live for the sake of living. Therefore, making female rural-to-urban migrant workers to be aware of their work is an urgent issue worthy of in-depth study.

The heterogeneous impacts of work value awareness on rural-to-urban migrant workers’ self-rated physical health of different generations are also investigated in this paper. The older generation rural-to-urban migrant workers’ work value awareness impacts their self-rated physical health more significantly than the younger generation. One possible reason for this result is that the younger generation rural-to-urban migrant workers may generally believe their physical health is in good condition. It is also the reality. Therefore, the homogeneity of the explained variable may lead the work value awareness to not correlate with the younger generation rural-to-urban migrant workers’ self-rated physical health. On the contrary, the older generation rural-to-urban migrant workers may have richer life experience and may rate their physical health more accurately than the younger generations. As such, the work value awareness impacts the older generations’ self-rated physical health significantly.

In the mechanism analysis, this paper finds that the rural-to-urban migrant workers’ mental health plays a mediating role in the impact of rural-to-urban migrant workers’ work value awareness on their self-rated physical health. Previous studies have indicated that individual physical health and mental health are highly correlated with each other [26,56]. Mental health is both a motivator and a barrier to physical health. There are two sides to the effect of mental health on physical health: positive and negative sides. On the positive side, some psychotherapy focusing on mental health can even cure some physical diseases that cannot be cured by certain drugs [57]. On the negative side, mental distress often deteriorates physical health via organic lesions [58]. Further, the five dimensions of work value awareness, including emotional, social, respect, ability, and interest value awareness, are based on the relative higher psychological hierarchy in HTN. As such, the work value awareness impacts workers’ mental health first, then it affects physical health afterward. However, in the group of rural-to-urban migrant workers, mental health or psychotherapy is an article of luxury [59]. The differential welfare policy setting centered on the registration system in China, the separated life mode far away from hometown and family, and the identity dilemma as immigrants in cities are the main factors that cause the mental health problems of rural-to-urban migrant workers [60]. Overall, to enhance the physical health of this group, mental health problems are the obstacle that must be eliminated. As this paper has demonstrated, work value awareness can play a vital role to achieve that goal.

## 6. Limitations

Still, there are some limitations to this paper. First, a more robust study can be carried out to see the dynamic changes of the impact of work value awareness on the health of workers, so as to draw a more robust conclusion. This is only possible with panel data. Second, work value awareness is a complex concept. If future research can develop a more detailed scale, it may help us to have a clear understanding of work value awareness and its various effects on workers. Third, we did not consider the acculturation of rural-to-urban migrants to the city environment due to data shortcomings. Acculturation can help migrants and other environmental program participants to accept new choices [61].

## 7. Conclusions

Using the national representative survey (CLDS2016) data, we explored the relationship between the awareness of work value of Chinese rural-to-urban migrant workers and their physical health. The awareness of work value was characterized by the awareness of emotional value, social value, respect value, ability value and interest value. Physical health was measured by self-rated health. The endogeneity issue was addressed by using an instrumental variable approach. The results showed that the awareness of work value impacts the physical health of rural-to-urban migrant workers significantly; mental health had mediated the impact of work value awareness on the physical health of rural-to-urban migrant workers. The results also showed that the impacts of work value awareness on rural-to-urban migrant workers’ physical health are heterogeneous by gender and age groups.

The United Nations sustainable development goals (SDGs) put forward 17 global development goals to be achieved by 2030. These goals include good health and well-being, as well as decent work and economic growth. Rural-to-urban migrant workers provide rich labor resources for China’s economic development; their health and well-being should be paid attention to. Rural-to-urban migrant workers’ awareness of work value is not only an important part of decent work awareness, but also affects their health. Therefore, it is essential to enhance rural-to-urban migrant workers’ awareness of work value, which is related to individual dignity, development, and well-being. In addition to China, there are many migrant workers around the world [62]. The findings of this study are applicable to other developing and developed countries as well.

## Figures and Tables

**Table 1:** Polychoric correlation coefficient test on variables of work value awareness.

Variable	Emotional Value Awareness	Social Value Awareness	Respect Value Awareness	Ability Value Awareness	Interest Value Awareness
Emotional value awareness	1				
Social value awareness	0.3543	1			
Respect value awareness	0.4075	0.4017	1		
Ability value awareness	0.3773	0.5513	0.6592	1	
Interest value awareness	0.4017	0.5176	0.5707	0.6930	1

Note: Response to each variable is measured on a Likert scale, with values ranging from 1 (strongly agree) to 5 (strongly disagree).

**Table 2:** Descriptive statistics.

Variable	Mean	SD	Min	Max
Self-rated physical health	3.82	0.86	1	5
Work value awareness	13.03	3.78	5	25
Emotional value awareness	2.38	0.97	1	5
Social value awareness	2.63	1.03	1	5
Respect value awareness	2.59	0.94	1	5
Ability value awareness	2.64	0.10	1	5
Interest value awareness	2.79	1.02	1	5
Age (year)	43.01	10.59	20	65
Education (year)	8.56	2.73	0	12
Income (CNY per year)	41,423.56	42,802.68	300	500,000
Mental health	25.79	7.48	20	76
**Variable**	**Item**	**Freq.**	**Percent**	
Gender	Male	480	52.86	
Marital status	Single	141	15.53	
	Married	757	83.37	
	Divorced	8	0.88	
	Widowed	2	0.22	
Religion	Western	18	1.98	
	Eastern	73	8.04	
	No religion	817	89.98	
Smoking	Yes (“1”)	286	31.50	
Drinking	Yes (“1”)	220	24.23	
*n*		908	100	

**Table 3:** Influences of rural-to-urban migrant workers’ work value awareness on their self-rated physical health (ordered probit model and IV-ordered probit model).

**Variable**	**Ordered Probit Model**			**IV-Ordered Probit Model**	
	(1)	(2)	(3)	(4)	(5)
Work value awareness	−0.0427 ***	−0.0425 ***	−0.0327 ***	−0.1671 **	−0.0512 *
	(0.0095)	(0.0097)	(0.0099)	(0.0799)	(0.0285)
Gender (Male)			0.1567	0.1056	0.1511
			(0.0996)	(0.1087)	(0.0999)
Age			−0.0257 ***	−0.0185 **	−0.0255 ***
			(0.0046)	(0.0073)	(0.0047)
Education			0.0050	−0.0044	0.0049
			(0.0151)	(0.0143)	(0.0151)
**Marital status (Single as reference)**					
Married			0.2497 **	0.1905	0.2482 **
			(0.1242)	(0.1277)	(0.1242)
Divorced			0.7876 *	0.9026 **	0.8389 *
			(0.4438)	(0.4234)	(0.4496)
Widowed			−1.5848 *	−0.8950	−1.5089 *
			(0.8377)	(1.0006)	(0.8442)
**Religion (Western religion as reference)**					
Eastern religion			0.1728	−0.1159	0.1514
			(0.3001)	(0.2913)	(0.3017)
No religion			0.1863	−0.0326	0.1819
			(0.2692)	(0.2535)	(0.2692)
Income			0.0176	−0.0098	0.0140
			(0.0306)	(0.0371)	(0.0311)
Smoking			−0.0525	−0.0931	−0.0566
			(0.1018)	(0.0995)	(0.1019)
Drinking			0.0315	0.0288	0.0293
			(0.0976)	(0.0940)	(0.0976)
Mental health			−0.0425 ***	−0.0289 **	−0.0415 ***
			(0.0052)	(0.0127)	(0.0054)
Region	No	Yes	Yes	No	Yes
Pseudo R^2^	0.0090	0.0306	0.0853		
*n*	908				

Notes: Standard errors in parentheses; *** *p* < 0.01, ** *p* < 0.05, * *p* < 0.1. “Yes” means the variable is added in the model.

**Table 4:** Impacts of rural-to-urban migrant workers’ work value awareness on their self-rated physical health on different groups (IV-ordered probit model).

Variable	Male	Female	Younger Generation	Older Generation
Work value awareness	−0.0394 *	−0.0094	0.0089	−0.0329 **
	(0.0216)	(0.0207)	(0.0395)	(0.0164)
Gender			0.0786	0.1217
			(0.1139)	(0.0873)
Age	−0.0147 ***	−0.0220 ***	−0.0094	−0.0160 ***
	(0.0043)	(0.0049)	(0.0135)	(0.0048)
Education	0.0237	−0.0067	0.0363	−0.0039
	(0.0172)	(0.0146)	(0.0273)	(0.0120)
**Marital status (Single as reference)**				
Married	0.1008	0.1523	0.1242	0.0219
	(0.1059)	(0.1257)	(0.1199)	(0.1875)
Divorced	0.7525 **	0.0403	0.6528 **	0.4633
	(0.2998)	(0.3262)	(0.3209)	(0.4055)
Widowed		−0.7945		−1.0873 *
		(0.9233)		(0.6158)
**Religion (Western religion as reference)**				
Eastern religion	0.0313	0.1747	0.6760 **	−0.0957
	(0.2604)	(0.3751)	(0.2950)	(0.2726)
No religion	−0.0593	0.3226	0.5277 **	0.0249
	(0.2344)	(0.3348)	(0.2478)	(0.2403)
Income	0.0025	0.0164	0.0211	0.0030
	(0.0373)	(0.0290)	(0.0304)	(0.0320)
Smoking	−0.0197	−0.9188 ***	0.1318	−0.1101
	(0.0723)	(0.2455)	(0.1187)	(0.0907)
Drinking	0.0994	−0.1008	−0.1117	0.1239
	(0.0746)	(0.2043)	(0.1383)	(0.0876)
Mental health	−0.0304 ***	−0.0299 ***	−0.0188 ***	−0.0352 ***
	(0.0048)	(0.0047)	(0.0054)	(0.0041)
Region	Yes	Yes	Yes	Yes
*n*	480	427	282	625

Notes: Standard errors in parentheses; *** *p* < 0.01, ** *p* < 0.05, * *p* < 0.1. “Yes” means variable is added in the model.

**Table 5:** Mediating effect of mental health in the impact of rural-to-urban migrant workers’ work value awareness on their self-rated physical health.

Model Pathways	Estimated Effect	95% CI	
		Lower Bounds	Upper Bounds
**Direct effect**			
Work value awareness → Physical health	−0.0056 ** (0.0023)	−0.0105	−0.0011
**Indirect effect**			
Work value awareness → Mental health → Physical health	−0.0242 *** (0.0079)	−0.0396	−0.0095

## Data Availability

Restrictions apply to the availability of these data. Data were obtained from the Center for Social Science Survey at Sun Yat-sen University in Guangzhou, China and are available from cssdata@mail.sysu.edu.cn with the permission of the Center for Social Science Survey at Sun Yat-sen University in Guangzhou, China.

## Appendix A

Center for Epidemiologic Studies Depression Scale (CES-D).

The 20 items below refer to how an individual has felt and behaved during the last week. Every item has the same selective answers including rarely or none of the time (<1 day), some or a little of the time (1–2 days), occasionally or a moderate amount of the time (3–4 days), most or all of the time (5–7 days).

1. I was bothered by things that do not usually bother me.
2. I did not feel like eating; my appetite was poor.
3. I felt that I could not shake off the blues even with the help of my family or friends.
4. I felt that I was just as good as other people.
5. I had trouble keeping my mind on what I was doing.
6. I felt depressed.
7. I felt everything I did was an effort.
8. I felt hopeful about the future.
9. I thought my life had been a failure.
10. I felt fearful.
11. My sleep was restless.
12. I was happy.
13. I talked less than usual.
14. I felt lonely.
15. People were unfriendly.
16. I enjoyed life.
17. I had crying spells.
18. I felt sad.
19. I felt that people disliked me.
20. I could not get “going”.
